# WHO Multidrug Therapy for Leprosy: Epidemiology of Default in Treatment in Agra District, Uttar Pradesh, India

**DOI:** 10.1155/2015/705804

**Published:** 2015-02-01

**Authors:** Anil Kumar, Anita Girdhar, Joy Kumar Chakma, Bhuwneswar Kumar Girdhar

**Affiliations:** National JALMA Institute for Leprosy & Other Mycobacterial Diseases, Agra, India

## Abstract

*Aim.* To study the magnitude of default, time of default, its causes, and final clinical outcome. *Methods*. Data collected in active surveys in Agra is analyzed. Patients were given treatment after medical confirmation and were followed up. The treatment default and other clinical outcomes were recorded. *Results*. Patients who defaulted have comparable demographic characteristics. However, among defaulters more women (62.7% in PB, 42.6% in MB) were seen than those in treatment completers (PB 52.7% and MB 35.9%). Nerve involvement was high in treatment completers: 45.7% in PB and 91.3% in MB leprosy. Overall default rate was lower (14.8%) in ROM than (28.8%) in standard MDT for PB leprosy (*χ*_1_^2^ = 11.6, *P* = 0.001) and also for MB leprosy: 9.1% in ROM compared to 34.5% in MDT (*χ*_1_^2^ = 6.0, *P* = 0.015). Default rate was not different (28.8% versus 34.5%, *P* > 0.05) in both types of leprosy given MDT. Most patients defaulted at early stage of treatment and mainly due to manageable side effects. *Conclusion*. The default in standard MDT both for PB and MB leprosy was observed to be significantly higher than in ROM treatment. Most defaults occurred at early stage of treatment and major contribution of default is due to side effects like drowsiness, weakness, vomiting, diarrhea, and so forth, related to poor general health. Although about half of the defaulters were observed to be cured 2.2% in PB-MDT and 10.9% of MB-MDT developed disability. This is an issue due to default. Attempts are needed to increase treatment compliance. The use of specially designed disease related health education along with easily administered drug regimens may help to reduce default.

## 1. Introduction

Leprosy remains a major public health problem in many developing countries and a major global share is coming from India. The multidrug therapy (MDT) is an effective and a powerful tool in curing leprosy, especially when patients report early and start prompt treatment. Adherence to treatment and its successful completion are equally important. Unfortunately, due to a number of personal, psychosocial, economic, and medical and health service factors, a significant number of patients become irregular and default from MDT. The treatment adherence and completion vary widely globally: <70% in multibacillary leprosy and 90% in paucibacillary leprosy [1, 2]. Poor adherence has detrimental consequences including incomplete cure, persisting infectious sources, transmission to new susceptible and multidrug resistance, and also risk of developing disability or deformity [3].

The success of the current WHO key strategy for leprosy elimination (i.e., MDT regimen) depends largely on the efficiency of health care delivery services and patient compliance to schedule treatment. A high rate of noncompliance with the regimen has serious implications for the leprosy control program because it can set the stage for the emergence of drug resistance, eventually resulting in treatment failure and failure of the program [3, 4].

Research on drug compliance has indicated that if a patient understands his/her disease and its treatment well, he/she is more likely to be motivated to take the whole prescribed course of treatment properly. It is widely believed that the understanding and behavior of patients in relation to drug compliance are largely influenced by their socioeconomic condition and level of knowledge [3]. In 1981, a WHO study group recommended that multibacillary (MB) leprosy patients should be given multidrug therapy (MDT) for at least two years and, wherever possible, until skin-smear becomes negative [5]. To improve operational efficiency as well as to improve patient compliance in leprosy programs, easily administrated drug regimens like ROM may be helpful.

The data used in this study comes from several field surveys conducted in Agra district and this provides an opportunity to assess the treatment completion by leprosy patients and the extent of defaulting, its correlates, and reasons. The data also provide an opportunity to compare with the WHO-ROM (once a month dose schedule) with WHO-MDT.

## 2. Methods and Material

This study is designed based on data available from several field based projects undertaken in Agra district during 2001 to 2010. In these surveys, house to house examination was done to suspect leprosy. Once a suspect is identified, he/she was medically examined by a clinician and after confirmation was put on treatment. During these household surveys, 1353 PB leprosy cases with 1 to 5 skin lesions with/without 1 nerve were detected. 300 cases having single skin lesion without nerve involvement were part of a trial to test efficacy of adding [C]larithromycin with single dose of ROM. Since ROM or CROM treatment was given once a month under supervision, thus, no question of default arise. This data was published [6] and therefore not included in the present analysis.

Data included in the analysis is presented in Table 1. A total of 1346 cases were detected and 1296 were finally included in the analysis.

Although a well organised effort was made to follow up the patients every 6 months who completed treatment, an attempt was also made to follow up defaulters at the closure of study, who defaulted for various reasons and their clinical condition was assessed.

All patients were informed about benefit of continuing treatment and possible damage of incomplete treatment and their consent was taken for examination and treatment after informing about disease, disease type, its treatment, and the risk associated. The treatment was provided on monthly basis at the doorstep after the case confirmation by a medical doctor and patients were monitored for treatment completion and for any side effects. The reported side effects were recorded as reported by the patients and managed in consultation with the doctor.

A patient is classified as defaulter if the scheduled 6-month treatment for PB leprosy is not completed in 9 months and 12-month treatment for MB leprosy is not completed in 18 months [2]. A patient who showed signs of intending default was counseled and if not successful classified as default. The counseling included attempts to inform the patients about benefit of continuing treatment and possible damage of incomplete treatment. Although followup was done every 6 months for those who completed treatment for about 6 to 8 years, defaulters were visited at the end of the study to check the disease status. The difference in the groups is compared using *χ* ^2^ test of significance.

## 3. Results

Demographic and clinical characteristics at detection are as follows: Among 1053 PB patients in Agra district, 736 (69.9%) completed 6-month schedule treatment, whereas 271 (25.8%) defaulted and the remaining 46 (4.3%) did not start treatment (refused). In 293 MB patients, 195 (66.5%) completed 12-month scheduled treatment, 94 (32.1%) defaulted, and** 4 (1.4%)** refused treatment.

### 3.1. Paucibacillary (PB) Leprosy

Among PB leprosy cases, the mean age (SD) of patients who completed the scheduled 6-month treatment was 34.7 (16.8) years in comparison to 36.1 (17.1) in defaulters and 34.8 (17.6) in those who refused treatment. The difference in mean age was not significant. More female patients (62.7%) were observed among the defaulters or among those who refused (65.2%) treatment (*χ*_2_^2^ = 9.8, *P* = 0.008) than 52.7% female patients among those who completed the scheduled treatment. The median delay at detection was 18 months among those who completed treatment and 12 months in each of those who defaulted or refused treatment. The mean numbers of leprosy lesions were closely similar: 1.83 (1.08) in those who completed treatment and 1.79 (1.04) in those who defaulted or 1.63 (0.88) in those who refused treatment. However, a significantly large number (45.7%) of patients among those who completed treatment had thickened nerve in comparison to 39.9% among defaulters and 23.9% in those who refused treatment (*χ*_2DF_^2^ = 10.0, *P* = 0.007). About 1.8% of those who completed treatment had disability (Grade 1 = 0.7%, Grade 2 = 1.1%) in comparison to 1.8% among defaulters (Grade 1 = 1.1%, Grade 2 = 0.7%) and none among those who refused treatment (Table 2).

### 3.2. Multibacillary (MB) Leprosy

The average (SD) age of MB patients at detection was 43.3 (17.2) years. It was 41.6 (16.9) years in those who completed treatment, 46.4 (17.6) years in those who defaulted, and 48.3 (16.6) years among those who refused treatment. The difference was not significant. The percent of female patients was 38.2% among MB patients, 35.9% among those who completed treatment, 43.6% among defaulters, and 50.0% among those who refused treatment (*P* > 0.05). The median delay at detection of leprosy was 36 months among each of those who completed treatment and defaulters and 42 months in those who refused. The number of skin lesions in MB leprosy patients was above 10 and 89.8% had thickened nerves. The percent of thickened nerve was high (91.3%) among those who completed treatment, 87.2% among defaulters, and 75.0% in those who refused treatment. About 10% of the MB leprosy patients had been detected with Grade 2 deformity (Table 2).

### 3.3. Magnitude and Time of Default

Of the total 1007 PB leprosy patients, 134 were part of trial under ROM (rifampicin, ofloxacin, and minocycline) and the rest were on standard WHO-MDT. In patients treated with PB-ROM, 20 (14.8%) defaulted, significantly less than 28.8% in WHO-PB-MDT (*χ*_1DF_^2^ = 11.6, *P* = 0.001). Similarly, in MB patients, a small number of 22 ROM patients could then be recruited due to the fact that its supply was stopped by WHO and only 2 out of 22 (9.1%) defaulted in comparison to 34.5% in WHO-MB MDT. The default rate was significantly high (*χ*_1DF_^2^ = 6.0, *P* = 0.015) in MDT group (Table 3). The overall rate of default in leprosy patients given WHO-MDT was found to be high: 28.8% in PB-MDT and 34.5% in MB-MDT. This was not significantly different (*χ*_1DF_^2^ = 3.17, *P* > 0.05).

### 3.4. Cumulative Default Rate by Time

Of the total PB leprosy cases being analyzed, 13.7% (138/1007) defaulted at first month itself; this increased cumulatively to 26.9% by the 5th month of the total PB cases. Although 50.9% (138/271) of total default took place in the first month itself, in subsequent months, it slowed down, like about 20% during the 2nd or 3rd month, 5.2% in 4th month, and 3.7% in 5th month. The pattern of defaulting has been nearly similar in ROM and MDT groups of PB leprosy.

**Figure 1:**
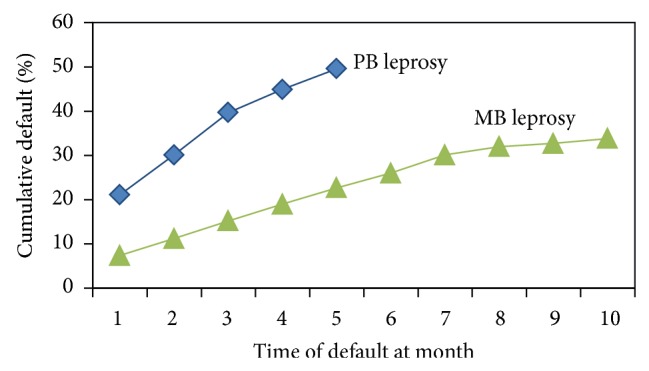
Cumulative default rate of treatment in PB/MB leprosy.

Similarly, of the 289 MB patients, 6.9% defaulted in the first month itself; it increased to 10.4% in 2nd month, increased to 14.2% by 3rd month, and further increased cumulatively to 32.5% by 11th month (Figure 1). The number of defaulters in the first 4 months was added to 54.3% (51/94) and subsequently the pace in defaulting number slowed down.

### 3.5. Cause of Default

#### 3.5.1. PB Leprosy (*N* = 271)

One of the most recorded complaints after the initiation of treatment was recorded in 50.9% (138/271) as intolerance or feelings of weakness/drowsiness followed by vomiting or diarrhea (10.0%) and complaint of swelling over the body (6.6%). Another 5.9% of patients discontinued multidrug treatment as they felt cured or were careless in taking treatment, 4.1% due to bad smell in drugs or inability to swallow the pills/capsules, and 2.6% due to bleeding in stool, and 2.6% felt as if it was just a scar on their skin and would automatically disappear. The second largest group (15.5%) of defaulters belonged to the group of “lost to treatment” due to either migration to other areas or job related nonavailability.

#### 3.5.2. MB Leprosy (*N* = 92)

The most important recorded complaint among MB patients was again intolerance/weakness/drowsiness/diarrhea in 60 patients (65.2%) of the MB-MDT group; some 9 cases (9.8%) discontinued treatment due to clofazimine/black face and other smaller problems and a few cases experienced reaction (4.3%), blood pressure/asthma (4.3%), and severe itching (2.1%) and unrelated deaths (2.1%) (Table 4). In whole MB group, excluding 2 cases of ROM, the pattern of default suggests that the highest (66%) is due to intolerance/weakness/drowsiness/diarrhea.

### 3.6. Clinical Outcome after Default

In 183 (67.5%) PB leprosy patients, the disease was cured subsequently after default and reported when they were last visited (average time = about 5 years after default); 8.5% remained with active skin lesions but no deformity. However, 3 developed Grade 1 disability (1 remained with active disease) and 3 developed Grade 2 deformity (1 with active disease) and 2 had type 1 reaction.

Among 36 (39.1%) of the MB patients on WHO-MDT, the disease was observed to have been cured; 17.4% continued with active disease but without deformity. A total of 10.9% of defaulters in MB-MDT group developed Grade 1 (2.2%) and Grade 2 (8.7%) deformity. Overall, in both types of leprosy, about 60% were observed to be cured after partial treatment or default, 10.7% remained status quo, and 4.3% developed disability Grade 1 or Grade 2 (Table 5).

## 4. Discussion

The treatment completion is however crucial to cure disease but several factors contribute to adherence to treatment schedule and to default. The side effects could range from mild to severe. It is well known that multidrug therapy (MDT) regimens can cause gastrointestinal problems, drowsiness, dizziness, and weakness [7]. A study observed that 62.9% of the patients in Brazil presented a low level of treatment adherence despite claiming to be aware of the risk of disease [8]. A study in Philippines showed 30% noncompliance rate of MDT treatment and causes for noncompliance were mainly drug related, health care provider-triggered, or patients induced or combinations of these [9]. Similarly, in Sudan, 40% of patients did not come to collect the complete treatment for leprosy [10]. In a retrospective assessment in Northern Mozambique between 1993 and 1997, 40.8% of them did not complete treatment [11].

The treatment default rate was found to be greater than 50% in New Delhi, India, 46% in TLM area, and 60% outside TLM area [12]. However, low treatment default rate of 3.4% was observed in a Brazilian study between 2001 and 2007 [13]. The default rate in the present study was found to be lower in ROM treatment of leprosy than that of standard MDT: for PB leprosy (14.8% versus 28.8%, *χ* ^2^ = 11.6, *P* = 0.001) and for MB leprosy (9.2% versus 34.5%, *χ* ^2^ = 6.0, *P* = 0.015). However, the default rate in standard MDT was found to be high both in PB (28.8%) and in MB leprosy (34.5%), respectively, but did not differ statistically. Time of default suggests that about half of defaulters do so in the first months. Although some of them could have developed very mild reaction which might have subsided within a day or two but was not reported by the patients.

In this study, more females were seen among the defaulters of PB leprosy and the reason could be their poor accessibility to health care facilities in their neighbourhood or their culture related dependence on male counterpart resulting in default. The severity of disease may also play a role as seen through the observation that patients who completed treatment had more nerve involvement and others have less nerve involvement. The possible risk of disability due to nerve involvement might have driven them to understand the need to complete treatment. However such a difference was not observed in MB leprosy. The reason could be that the majority of them had nerve involvement; thus pain is obvious. Therefore, no significance in difference was observed.

Interesting is to note that about half of the PB ROM and 2/3rd of PB-MDT defaulters were observed to have the skin lesions healed in spite of defaulting. Among the defaulters of MB leprosy in MB-MDT, 37% were observed to be cured (Table 5). However, the other side of observation is that 6 (2.2%) of the defaulters on PB treatment developed either Grade 1 or Grade 2 disabilities and in 8.5% disease could not be cured and thus remained status quo. Similarly, 10 (10.6%) of defaulters on WHO-MB-MDT treatment developed Grade 1 or Grade 2 disability and 17% of the defaulters were not cured from the disease.

The implication of the study is, therefore, the concern regarding the risk of developing disability that seems to be increasing due to default and this might also increase chances of transmission to other susceptables in the community depending upon the transmission potential of the untreated case of leprosy. Therefore, urgent steps are required to minimize default from treatment in the interests of affected patients and also for programme to create confidence by minimizing side effects. Well designed disease specific health education along with good health service facilities and information on available facilities and places may enhance utilization and thus help to reduce default.

## 5. Conclusion

The default rates were observed to be high in standard WHO-MDT treatment both for PB and for MB leprosy compared with in ROM treatment in this study. Most defaults occurred at early stage of treatment and major contribution of default is due to manageable side effects like drowsiness, weakness, vomiting, diarrhea, and so forth, which could be due to factors like poor general health. However, it was also observed that many defaulters were observed to have been cured at longer followup but 2.2% of the defaulters in PB leprosy and 10.9% of WHO-MB-MDT developed disability. This is a cause of concern due to default. Attempts need to be made to minimize this factor through increasing treatment compliance.

## Figures and Tables

**Table 1:** Details on inclusion of cases in the study.

	PB leprosy	MB leprosy	Total cases of leprosy

Number of cases detected	1053	293	1346
Refused treatment	46	04	50
In the study	1007	289	1296
ROM	34	22	
MDT	873	267	

**Table 2:** Demographic and clinical data at detection.

Characteristics	PB leprosy (1053)				MB leprosy (293)			
	TT done (736)	Defaulters (271)	Refused (46)	Total (1053)	TT done (195)	Defaulters (94)	Refused (4)	Total (293)
Mean age (SD)	34.7 (16.8)	36.1 (17.1)	34.8 (17.6)	35.1 (16.9)	41.6 (16.9)	46.4 (17.6)	48.3 (16.6)	43.3 (17.2)
Female (%)	52.7	62.7	65.2		35.9	42.6	50.0	38.2
Median delay in detection (months)	18	12	12		36	36	42	36
Mean (SD) number of skin lesions	1.83 (1.08)	1.79 (1.04)	1.63 (0.88)		13.5 (17.4)	14.6 (18.3)	12.3 (6.3)	13.8 (17.5)
(%) Nerve involvement	45.7	39.9	23.9		91.3	87.2	75.0	89.8
Deformity (%) Grade 1 (0.8) Grade 2 (1.0)	0.7 1.1	1.1 0.7	0 0		— 10.8	— 9.6	— 0	— 10.2

(1) ANOVA reflect no mean is significant (NS)					(1) ANOVA reflect no mean is significant			
(2) Nerve % involvement, *χ*_2DF_^2^ = 10.0. *P* = 0.007					(2) Nerve % involvement, NS			
(3) Disability NS					(3) Disability NS			
(4) Percent female, *χ*_2DF_^2^ = 9.8. *P* = 0.008					(4) Percent female, NS			

**Table 3:** Magnitude of default (%) by treatment type.

Treatment type	PB leprosy		MB leprosy	
	*N*	% default	*N*	% default
ROM	134	14.8	22	9.1
MDT	873	28.8	267	34.5
Total	**1007**	**26.9**	**289**	**32.5**
*χ*_1DF_^2^, *P* value	11.6, 0.001		6.0, 0.015	

**Table 4:** Causes of default.

Causes	PB leprosy			MB leprosy		
	PB-ROM	PB-MDT	% total (*n*)	MB-ROM	MB MDT	% total (*n*)
Complaint of heat/weakness	10	128	50.9 (138)	2	60	66.0 (62)
Complaint of swelling all the body	2	16	6.6 (18)			
Vomiting/diarrhea/acidity	4	24	10.3 (28)			
Feeling cured/carelessness	0	16	5.9 (16)			
Bad smell/unable to swallow	0	11	4.1 (11)			
Bleeding	0	7	2.6 (7)	0	3	3.2 (3)
Feeling of having no leprosy	0	7	2.6 (7)			
Stigma, leprosy related	0	2	0.7 (2)			
Migrated	4	38	15.5 (42)	0	9	9.6 (9)
Refused for pregnancy	0	1	0.4 (1)			
Reaction	0	1	0.4 (1)	0	4	4.2 (4)
Unrelated death				0	2	2.1 (2)
Blackish face (clofazimine)				0	7	7.5 (7)
Severe itching					2	2.1 (2)
High BP/asthma					4	4.3 (4)
Changed to medical college					1	1.0 (1)

Total	20	251	100.0 (271)	02	92	100.0 (94)

**Table 5:** Outcome events at “last visit to patient” after default.

Outcome at last visit	Type of treatment				
	PB-ROM	PB-MDT	MB MDT	MB-ROM	Total
Cured	50.0 (10)	68.9 (173)	37.0 (34)	100.0 (2)	60.0 (219)
Not cured, no deformity	5.0 (01)	8.8 (22)	17.4 (16)	0	10.7 (39)
Grade 1 disability	0	0.8 (2)	1.1 (01)	0	0.8 (03)
Grade 2 deformity	0	0.8 (2)	6.5 (06)	0	2.2 (08)
Reaction	5.0 (01)	0.4 (1)	1.1 (01)	0	0.8 (03)
Died	0	3.2 (8)	8.7 (08)	0	4.4 (16)
LFU	35.0 (07)	16.7 (42)	25.0 (23)	0	19.7 (72)
Active + Grade 1 disability	0	0.4 (1)	1.1 (01)	0	0.5 (02)
Active + Grade 2 disability	5.0 (01)	0	2.2 (02)	0	0.8 (03)

Total	100.0 (20)	100.0 (251)	100.0 (92)	100.0 (02)	100.0 (365)
